# Suppressive effects of long-term exposure to *P*-nitrophenol on gonadal development, hormonal profile with disruption of tissue integrity, and activation of caspase-3 in male Japanese quail (*Coturnix japonica*)

**DOI:** 10.1007/s11356-015-4245-9

**Published:** 2015-03-15

**Authors:** Eman Ahmed, Kentaro Nagaoka, Mostafa Fayez, Mohamed M. Abdel-Daim, Haney Samir, Gen Watanabe

**Affiliations:** 1 Laboratory of Veterinary Physiology, Department of Veterinary Medicine, Tokyo University of Agriculture and Technology, 3-5-8 Saiwai-cho, Fuchu, Tokyo 183-8509 Japan; 2 Department of Pharmacology, Faculty of Veterinary Medicine, Suez Canal University, Ismailia, 41522 Egypt; 3 Department of Theriogenology, Faculty of Veterinary Medicine, Cairo University, Giza, 12211 Egypt

**Keywords:** Caspase-3, Claudin-1, Diesel exhausts particles, Endocrine disrupting compounds, Hypothalamic–pituitary axis, Japanese quail, *P*-nitrophenol

## Abstract

*P*-Nitrophenol (PNP) is considered to be one of nitrophenol derivatives of diesel exhaust particles. PNP is a major metabolite of some organophosphorus compounds. PNP is a persistent organic pollutant as well as one of endocrine-disrupting compounds. Consequently, bioaccumulation of PNP potentiates toxicity. The objectives of the current study were to assess in vivo adverse effects of long-term low doses of PNP exposure on reproductive system during development stage. Twnety-eight-day-old male Japanese quails were orally administered different doses of PNP (0, 0.01, 0.1, 1 mg/kg body weight) daily for 2.5 months. Testicular histopathology, hormones, caspase-3 (CASP3), and claudin-1 (CLDN1) tight junction protein, as well as plasma hormones were analyzed. The results revealed that long-term PNP exposure caused testicular histopathological changes such as vacuolation of spermatogenic cell and spermatocyte with significant testicular and cloacal gland atrophy. PNP activated CASP3 enzyme that is an apoptosis-related cysteine peptidase. Besides, it disrupted the expression of CLDN1. Furthermore, a substantial decrease in plasma concentrations of luteinizing hormone (LH) and testosterone was observed after 2 and 2.5 months in the PNP-treated groups. Meanwhile, the pituitary LH did not significantly change. Site of action of PNP may be peripheral on testicular development and/or centrally on the hypothalamic–pituitary–gonadal axis through reduction of pulsatile secretion of gonadotrophin-releasing hormone. Consequently, it may reduce the sensitivity of the anterior pituitary gland to secrete LH. In conclusion, PNP induced profound endocrine disruption in the form of hormonal imbalance, induction of CASP3, and disruption of CLDN1 expression in the testis. Hence, it may hinder the reproductive processes.

## Introduction

There is an increase in public and scientific communities’ attention concerning the effects of endocrine-disrupting chemicals on the reproductive and the endocrine systems. It is well known that the decline in human, livestock, and wildlife endocrine health is mainly caused by environmental pollution (Stone 1994). Many environmental chemicals have been experimentally demonstrated that they adversely affect the endocrine processes (Zhang et al. 2013), known as endocrine disruptors. Endocrine disrupting chemicals are endocrine-modifying substances that have weak intrinsic hormonal or anti-hormonal activity and affect the balance of normal hormonal functions when those chemicals enter the body through ingestion or absorption (Keith 1998; Sharpe and Irvine 2004; Stone 1994; Zhang et al. 2013). Exposure to endocrine disruptors, specifically during developing stages, may cause dysfunctions or abnormalities of the reproductive organs later in adulthood (Timms et al. 2005). Diesel exhaust particles (DEPs) have an environmental and occupational health concern (Taneda et al. 2004). DEPs have become one of the main factors incriminated in various hazardous health problems for instance, reproductive dysfunction (Taneda et al. 2004), lung cancer (Ichinose et al. 1997), and bronchial asthma-like disease (Sagai et al. 1993).

*P*-Nitrophenol (PNP) is not only one of nitrophenol derivatives of DEPs (Noya et al. 2008) but also an intermediate chemical substance used in the manufacture of drugs, fungicides, rubber materials, and dyes (ATSDR 1992). Furthermore, PNP is a major metabolite of organophosphorus insecticides (Benke and Murphy 1975; Li et al. 2006a) such as parathion, fenitrothion, and methyl parathion, which are widely used pesticides, acaricide, and pre-harvest treatment in outdoors as well as in the greenhouse systems worldwide (Abu-Qare et al. 2000; Kim et al. 2006). PNP is considered as EDCs because it has an adverse impact on both male and female reproductive functions (Li et al. 2006a, b, 2009; Yue et al. 2011). PNP exhibits estrogen-like effects on female rats and anti-androgen-like effects on male rats (Li et al. 2006a). PNP is commonly found in water and soil from agriculture and industrial manufacturing (Bhushan et al. 2000). Therefore, potential for exposing humans, livestock, and wild animals to the PNP through these resources is very high. Consequently, US Environmental-Protection Agency rated nitrophenol as a priority pollutant (as HR-3 grade) and recommended to restrict its concentration in the natural water below10 ng ml^−1^ (US-EPA 1976).

The Japanese quail (*Coturnix japonica*) is considered an ideal biological and experimental model due to its fast development. Quails reach sexual maturity at 6–7 weeks old (Ball and Balthazart 2010; Sedqyar et al. 2008). In the maturing male, testosterone concentrations, testicular weight, and cloacal gland area increase dramatically between 26 and 35 days of age then increase rapidly until sexual maturity. Quails are representative for terrestrial birds and an accepted model for assessing both the acute and chronic effects of pesticides and other chemicals (OECD 1993; US-EPA 1996). They were used to examine the toxic effect of PNP by two reasons. Firstly, PNP is metabolized by cytochrome *P450* enzymes (Abu-Qare et al 2000; Machida et al. 1982). Cytochrome *P450* enzymes level in humans, chicken, and quail livers is not high, so they have lower catalytic activity than rats. Therefore, the compound stays for a longer period in the body resulting in greater accessibility to target tissues (Abu-Qare et al. 2000; 2001; Hansen et al. 2011). Hence, quails are more sensitive to toxicity compared with rats. Therefore, both bioaccumulation of PNP and moderate rate of clearance potentiate PNP toxicity. Secondly, quails have well-developed neuroendocrine systems that share fundamental properties with other vertebrate species including mammals (Ball and Balthazart 2010; Wingfield 2005).

Despite the potential significant toxic effects, basic data on the toxicity of PNP are very rare. Furthermore, there is a lack of knowledge about the extent to which a long-term low dose of PNP exposure affects the reproductive health condition, especially when there is a large difference between high exposures reported in laboratory experiments and the relatively low levels found in the environment (Vandenberg et al. 2009). In this vein, the present study was designed to identify the basic potential reproductive problems associated with PNP. We used different low doses of PNP with long-term exposure (2.5), especially during the development stages to clarify the basic potential endocrine and reproductive dysfunction due to PNP exposure.

For this purpose, male Japanese quails were used as a laboratory animal to elucidate the in vivo toxic effects of PNP on reproductive function, puberty, gonadal development, and interrelated hormonal changes. Moreover, the effects on testicular tissue architecture, CASP3 enzyme expression, and CLDN1 tight junction protein expression (distribution and staining intensity) were examined.

## Materials and methods

### Chemicals and primary antibodies

*P*-Nitrophenol crystal (4-nitrophenol; PNP, CAS No. 100-02-7, C_6_H_5_NO_3_, >99.9 % purity, molecular weight at 139.11 g/mol) was used in this experiment (Tokyo Kasei Kogyo Co. Ltd, Tokyo, Japan). Rabbit caspase-3 and rabbit claudin-1 antibodies were purchased from Cell Signaling Technology Co. Japan. These antibodies were used for tissue immunohistochemistry.

### Birds

Male Japanese quails (28-day-old and weighed 80–90 g) were housed in metal cages in a controlled environment (lights on, 0500–1900 hours; temperature, 24 ± 2 °C; humidity, 50 ± 10 %, and air exchange 20 times hourly) were used in this study. Birds were provided with food (Kanematsu quail diet; Kanematsu Agri-tech Co. Ltd, Ibaraki, Japan) and water ad libitum. Birds were treated humanely with regard to alleviating birds suffering. This study was conducted in accordance with the guideline principles established by Tokyo University of Agriculture and Technology, for use of laboratory animals. The protocol was approved by the Committee of the Ethics of animal experiments of Tokyo University of Agriculture and Technology.

### Experimental design

#### Administrations of PNP

Quails were randomly divided into four groups (*n* = 25–26 each) and assigned to the following treatments: (i) control group (phosphate-buffered saline), (ii) PNP low dose (0.01 mg/kg b.w.), (iii) PNP mid-dose (0.1 mg/kg b.w.), and (iv) PNP high dose (1.0 mg/kg b.w.). The PNP was dissolved in 0.01 M phosphate-buffered saline (PBS; pH 7.2) and administered to quail daily for 2.5 months using plastic stomach tube. The doses were decided depending on PNP pharmacokinetics (Abu-Qare et al. 2000; Machida et al. 1982) and from previous studies (Li et al. 2006a, b, 2009; Zhang et al. 2013).

#### Tissue sampling, testes weights, and cloacal measurement

Five quails per group were weighed then euthanized by decapitation at different time intervals as follows 0.5, 1, 1.5, 2, and 2.5 months post-treatment. Jugular blood samples were collected in heparinized plastic tubes and centrifuged at 1700×*g* for 15 min at 4 °C. Plasma was separated and stored at −20 °C until the hormonal assay was conducted. Testes and anterior pituitary gland were collected. The right and left testes were weighed separately. The cloacal gland areas (the longest length × the greatest width) were measured (Li et al. 2006b).

#### Tissue preparation

The anterior pituitary gland and one testis were washed in ice-cold saline and kept in 1 ml physiological saline (0.9 % NaCl). The pituitary and testes were sliced into small pieces, and then homogenized in 1 ml physiological saline. The homogenates were centrifuged at 20,000×*g* for 30 min at 4 °C. The supernatants were collected and stored at −20 until hormonal assay was conducted. The other testis was fixed for 24 h in 4 % paraformaldehyde in PBS at pH 7.4 (Sigma-Aldrich Chemical, ST. Louis, MO, USA) and kept in 70 % ethanol for histopathology and immunohistochemistry.

### Hormonal assay

Hormonal assay was performed using double-antibody radioimmunoassay (RIA) system using ^125^I-labeled radioligands in triplicates. Plasma and pituitary homogenate concentrations of LH were measured with a USDA-ARS radioimmunoassay (RIA) kit (Beltsville, MD, USA) for chicken LH. The antiserum used was anti-avian LH (HAC-CH27-01RBP75 in NRS). ^125^I-chicken LH (USDA-cLH-I-3) was used for iodination (Krishnan et al. 1994). LH radioimmunoassay materials and antiserum against LH were kindly provided by Dr JA Proudman, USDA-ARS, Biotechnology and Germplasm Laboratory (Beltsville, MD, USA) and the Biosignal Research Center, Institute for Molecular and Cellular Regulation, Gunma, Japan. Plasma concentrations of testosterone (Taya et al. 1985) and corticosterone (Kanesaka et al. 1992) were determined by double-antibody RIA system using ^125^I-labeled radioligands. The antiserum against testosterone was sheep anti-testosterone (GDN250), which kindly provided by Dr. GD Niswender, Animal Reproduction and Biotechnology Laboratory (Colorado State University, Fort Collins, CO, USA). The antiserum against corticosterone was goat anti-corticosterone. Plasma and testicular concentrations of immunoreactive (ir-)inhibin were measured by a double-antibody RIA, as described previously (Hamada et al. 1989). The antiserum used was rabbit antiserum against bovine inhibin (TNDH-1). Purified bovine at 32-kDa inhibin was used for iodination and standard.

### Histopathology

The fixed testes were dehydrated through a series-graded concentrations of ethanol, clarified in xylene, embedded in paraffin, sectioned at 4 μm, and placed on poly-l-lysine-coated slides (Sigma) for hematoxylin and eosin staining. Sections were examined by light microscopy. The criteria used for evaluation of histopathological changes of testes were followed from the principles described by Hess et al. (1988). An average number of 250 seminiferous tubules per animal (*n* = 4) were examined as reported by Thompson et al. (2009) and testicular degeneration was qualitatively assessed and scored into 4 scores as follows: normal (1), mild (2), moderate (3), and severe (4).

### Immunohistochemistry of caspase-3 and claudin-1

Testes section slides were deparaffinized with xylene and rehydrated in graded ethanol before being washed with distilled water for 5 min twice. The sections were treated with 3 % H_2_O_2_ to block endogenous peroxidase activity. Then, they were heated in (0.01 M) citrate buffer at 120 °C for 20 min in a microwave oven to retrieve antigen. The sections were cooled and washed three times with 0.01 M PBS, pH 7.2. Furthermore, they are blocked with goat normal serum in 0.01 M PBS for 30 min at room temperature. The antiserum-treated sections were incubated overnight at 4 °C with polyclonal antibodies against CASP3 and CLDN1, diluted at 1:500 and 1:1000, respectively. The second antibody (biotinylated anti-rabbit IgG) was added for 30 min, and then washed with PBS. After that, the horseradish peroxidase was added for 30 min. The bindings of the antibodies were visualized using the liquid DAB Substrate Kit (using diaminobenzidine as a chromogen substrate, Sigma). Tissue sections were counterstained with hematoxylin, dehydrated, cleared, and mounted with coverslips. The specificity of the antibody was examined using 1 % normal goat serum without the primary antibody. In regard to CASP3, the average number of positive cells was calculated in 30 fields/slide in five animals using magnification power ×200. Then, the data obtained were statistically analyzed.

## Statistical analysis

Data were tested for homogeneity distribution of variance using GraphPad Prism. The relative testes weight, cloacal gland areas, and hormonal changes were statistically analyzed using one-way analysis of variance (ANOVA). The significant differences were analyzed with Tukey’s multiple comparison tests at each time point in relation with the control group at the same time. The statistical analysis was performed using the software program GraphPad Prism version 5. All data were expressed as means ± S.E.M. A probability value of *P* < 0.05 was considered as significant. Moreover, the testis weight loss was expressed as percentage when compared with the control group at the same time. Four scores of testicular degeneration and caspase-3-positive cell expression were statistically analyzed using ANOVA followed by Tukey’s multiple comparison tests for significant difference.

## Results

### Effect of PNP on body weight and testicular development

**Fig. 1:**
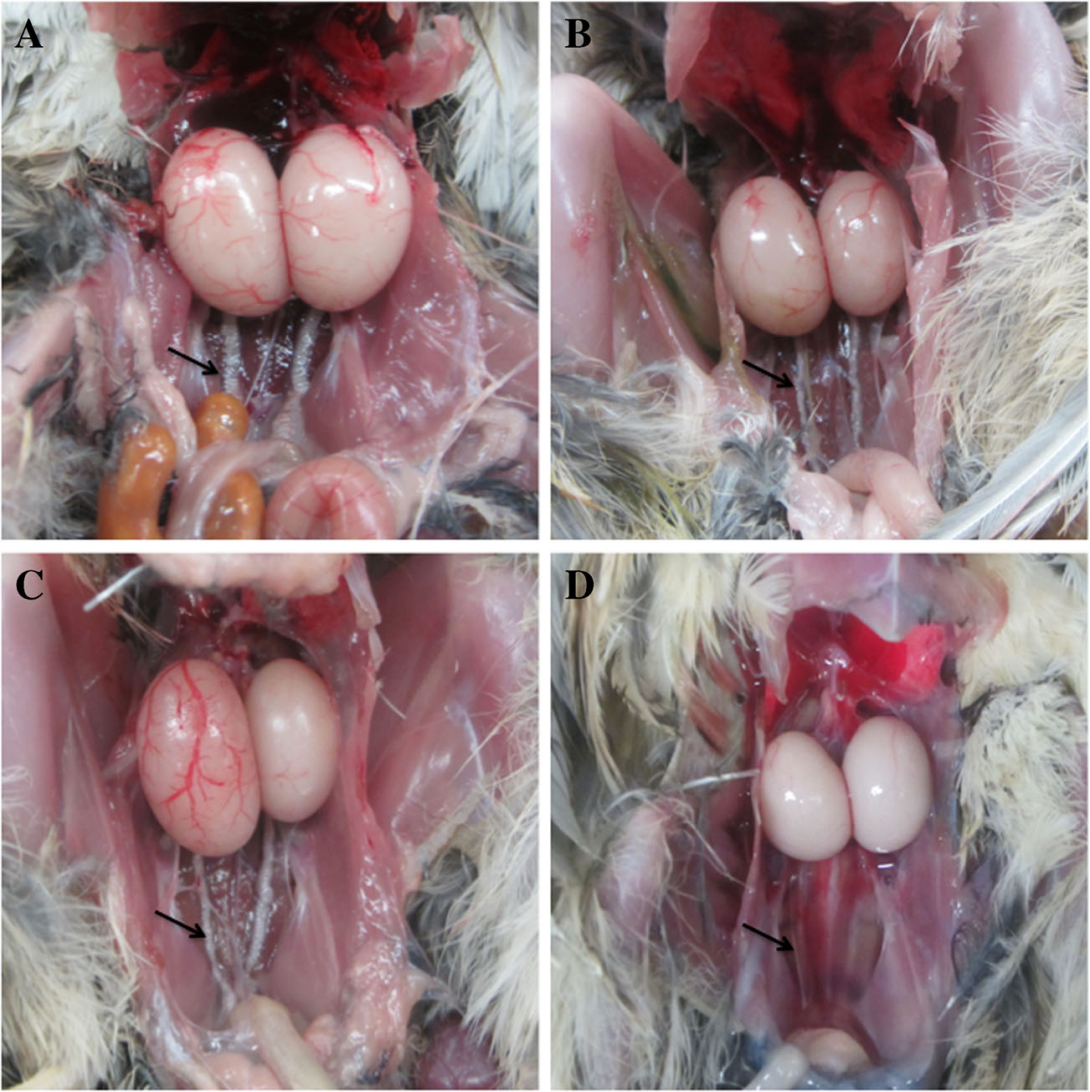
Gross examination of the testes in the control (**a**) quail with well vascularization and well-developed vas deferens (*arrow*) and unilateral and bilateral atrophied testes in the PNP-treated quails, mid-dose (**b**) and high (**c**, **d**), with less vascularization and ill-developed vas deferens (*arrow*)

Although, PNP administration for 2.5 months had no significant effect on the body weight gain (data not shown), it induced significant decrease in the testes weight and the degree of vascularization (Tables 1 and 2; Fig. 1). A high rate of relative testicular weight loss was observed at 2 months post-PNP exposure in the mid- and the highest-dose groups when compared with the control group (74.7 and 81 vs. 100 % for the right testes and 67.9 and 65.2 vs. 100 % for the left testes), respectively. Some birds showed bilateral testes atrophy, but others showed an asymmetrical unilateral decrease. The PNP treatment induced testicular atrophy, but neither the severity nor the incidence of the atrophy showed a dose-dependent relationship because both mid- and high doses showed the high degree of relative testicular weight loss and damage. Furthermore, ductus deferens was atrophied and not as well developed in the PNP-treated groups (mid- and high doses) as in the control group (Fig. 1).

**Table 1:** The relative weight of the right testis (g) in the control and the PNP-treated groups

Time (after PNP administration)		PNP (mg/kg b.w.)		
	Control (PBS)	0.01 (low dose)	0.1 (mid dose)	1.0 (high dose)
0.5 month	0.32 ± 0.13	0.25 ± 0.07	0.54 ± 0.12	0.40 ± 0.13
1 month	1.18 ± 0.12	1.34 ± 0.17	1.45 ± 0.20	1.16 ± 0.22
1.5 months	1.44 ± 0.08	1.54 ± 0.08	1.27 ± 0.17	1.46 ± 0.12
2 months	1.74 ± 0.08	1.49 ± 0.06	1.30 ± 0.05*	1.41 ± 0.07*
2.5 months	1.43 ± 0.17	1.31 ± 0.11	1.53 ± 0.15	1.33 ± 0.16

Each value represents the mean ± SEM of (*n* = 5) quails per group

*P < 0.05, significant difference (the PNP-treated groups vs. the control group)

**Table 2:** The relative weight of the left testis (g) in the control and the PNP-treated groups

Time (after PNP administration)		PNP (mg/kg b.w.)		
	Control (PBS)	0.01 (low dose)	0.1 (mid dose)	1.0 (high dose)
0.5 month	0.38 ± 0.15	0.26 ± 0.07	0.56 ± 0.16	0.63 ± 0.20
1 month	1.31 ± 0.15	1.47 ± 0.13	1.51 ± 0.09	1.32 ± 0.19
1.5 months	1.52 ± 0.20	1.47 ± 0.06	1.51 ± 0.19	1.60 ± 0.08
2 months	1.87 ± 0.17	1.79 ± 0.13	1.27 ± 0.18*	1.22 ± 0.10*
2.5 months	1.72 ± 0.13	1.83 ± 0.12	1.36 ± 0.09	1.59 ± 0.11

Each value represents the mean ± SEM of (*n* = 5) quails per group

*P < 0.05, significant difference (the PNP-treated groups vs. the control group)

### Effect of PNP on cloacal gland areas

**Fig. 2:**
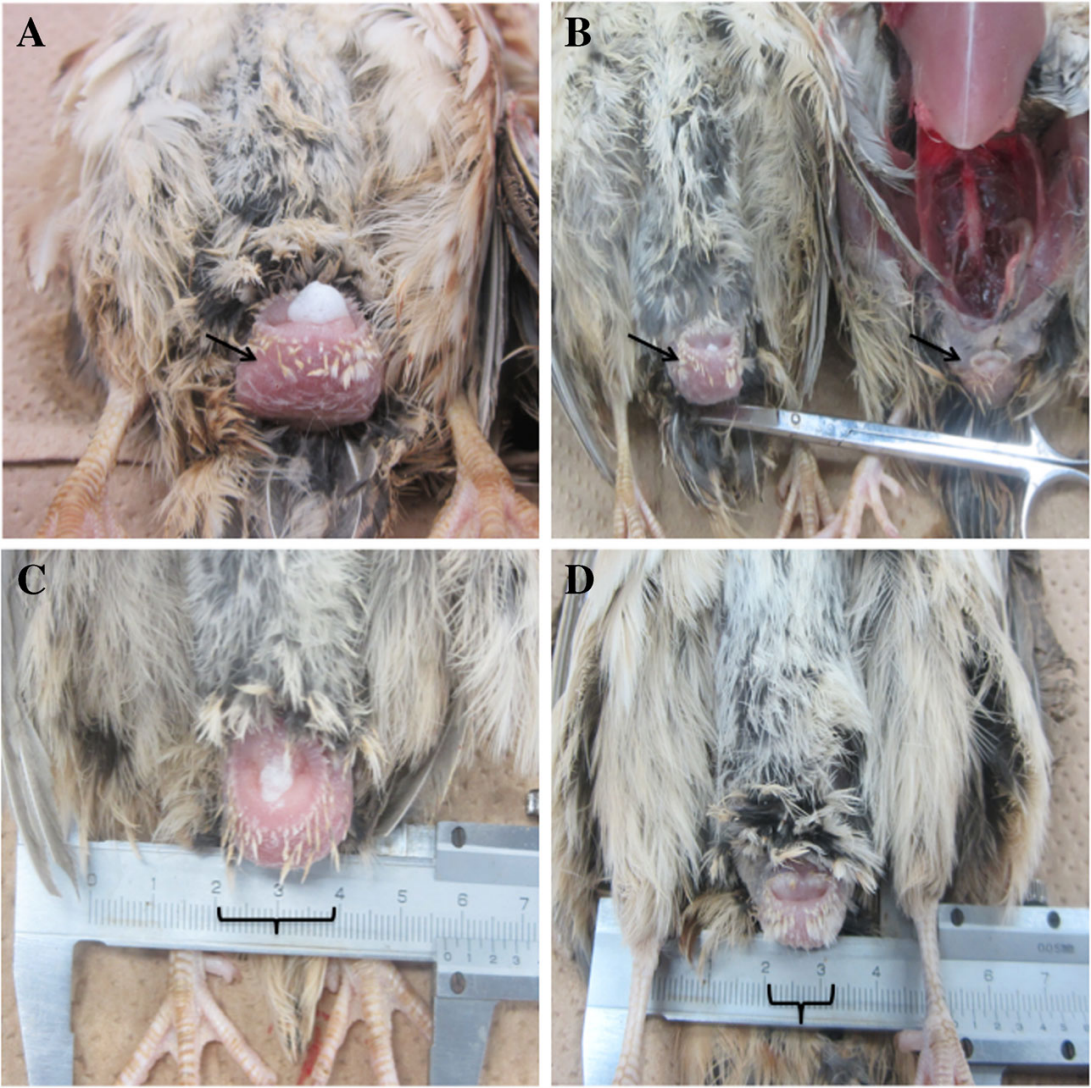
Cloacal glands in the control quails were well developed with foam formation (**a**, **c**) and atrophied cloacal gland in the PNP-treated quails (high (**b**) and mid-dose (**d**)) with less or no foam formation

Cloacal gland was highly affected by the toxicity of PNP. PNP caused a significant decrease in the cloacal gland area after 1.5, 2, and 2.5 months as shown in Table 3. After 2.5 months, exposure to PNP caused profound disparity in the treated groups vs. the control group (63.7, 31.4, and 43.1 vs. 100 %, respectively). The cloacal glands of the control birds had normal morphology with normal cloacal gland foam production (Fig. 2). On the other hand, the PNP-treated birds had smaller cloacal glands (Fig. 2a, c) and did not produce cloacal gland foam (Fig. 2b, d).

**Table 3:** The cloacal gland area in the control and the PNP-treated groups

Time (after PNP administration)		PNP (mg/kg b.w.)		
	Control (PBS)	0.01 (low dose)	0.1 (mid dose)	1.0 (high dose)
0.5 month	0.37 ± 0.07	0.38 ± 0.07	0.40 ± 0.08	0.60 ± 0.22
1 month	1.23 ± 0.10	0.93 ± 0.09	0.92 ± 0.13	0.90 ± 0.14
1.5 months	1.17 ± 0.16	1.01 ± 0.12	0.57 ± 017*	0.74 ± 0.05
2 months	1.29 ± 0.13	1.036 ± 0.10	1.026 ± 0.12	0.7814 ± 0.01**
2.5 months	1.02 ± 0.11	0.65 ± 0.10*	0.32 ± 0.11***	0.44 ± 0.04**

Each value represents the mean ± SEM of (*n* = 5) quails per group

*P < 0.05; **P < 0.01; ***P < 0.001, significant difference (PNP-treated groups vs. control group)

### Effects of PNP on circulating, pituitary, and testicular hormones

**Fig. 3:**
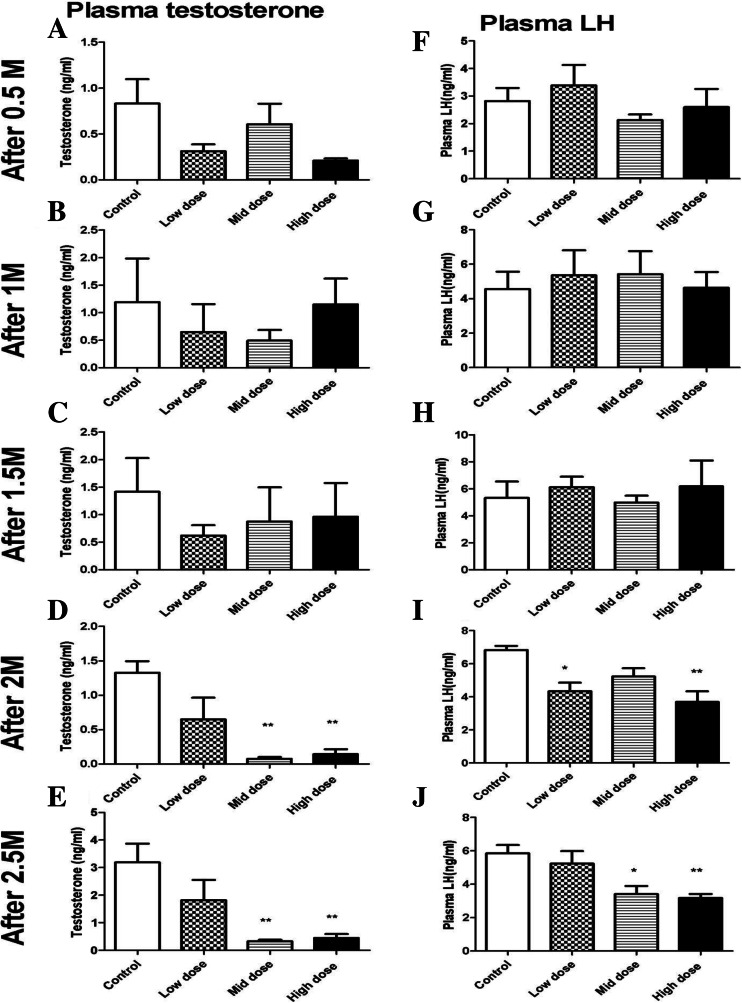
Plasma concentrations of the testosterone (**a**–**e**) and the LH (**f**–**j**) at the different times in the control and the PNP-treated Japanese quails (0.01, 0.1, or 1 mg/kg b.w.). *Each bar* represents the mean ± S.E.M. of five birds per group. *P < 0.05; **P < 0.01 compared with value for control quail

**Fig. 4:**
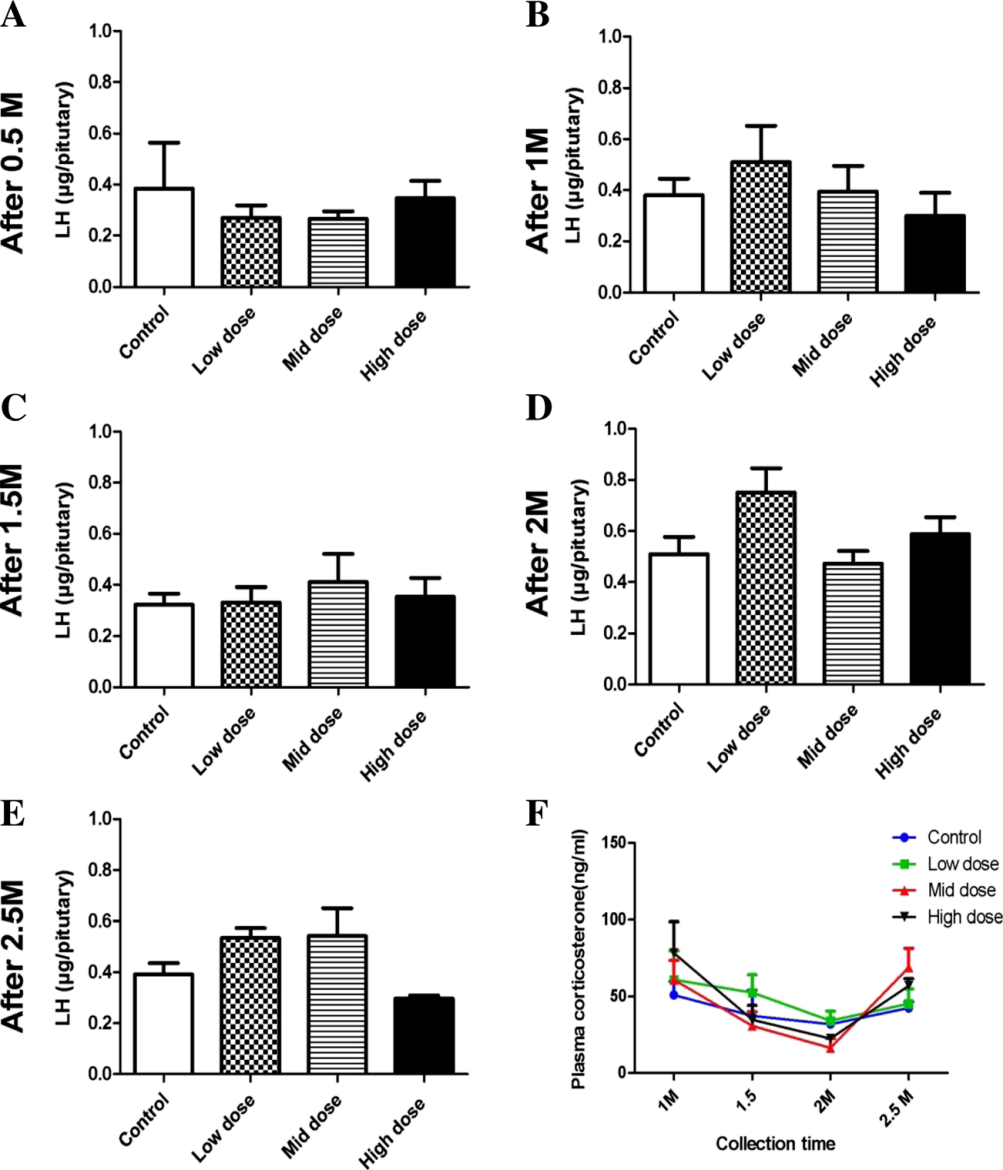
Pituitary LH (**a**–**e**) and plasma concentrations of corticosterone (**f**) at different times in the control and the PNP-treated Japanese quails (0.01, 0.1, or 1 mg/kg). *Each bar* represents the mean ± S.E.M. of five birds per group

Plasma concentrations of testosterone and LH in the PNP-treated birds showed significant decrease after 2 and 2.5 months as shown in Fig. 3. Plasma concentrations of the testosterone (Fig. 3a–e) were significantly lower in both the 0.1- and 1.0-mg/kg-treated groups when compared with the control group after 2 months (0.076 ± 0.02, 0.14 ± 0.07vs. 1.33 ± 0.17) and 2.5 months (0.33 ± 0.05, 0.44 ± 0.14 vs. 3.19 ± 0.68 ng/ml), respectively. Testosterone level starts to record nonsignificant decrease and fluctuation after 0.5 months of PNP exposure. Concurrently, plasma concentrations of LH were significantly lower in the low and the high dose (4.33 ± 0.25 and 3.69 ± 0.65 ng/ml, respectively) after 2 months when compared with the control birds (6.83 ± 0.25 ng/ml) (Fig. 3i). Furthermore, after 2.5 months, LH hormone was 3.40 ± 0.49 and 3.1 ± 0.24 in the mid- and the high group, respectively, when compared with 5.94 ± 0.5 for the control birds (Fig. 3j). The concentrations of LH in the pituitary homogenate after 2.5 months tend to be increased in the low and the mid-doses, meanwhile they tend to decrease in the highest dose of the PNP (Fig. 4a–e). Plasma corticosterone (Fig. 4f) did not significantly increase after 1 month in low, mid-, and high groups (61.80 ± 16.94, 60.87 ± 12.54, and 78.35 ± 17.29, respectively) when compared with the control group (50.98 ± 8.678). After 2 months of PNP administration, the plasma corticosterone tended to decrease in the mid- and the high-dose groups when compared with the control group (16.32 ± 6.2 and 22.48 ± 2.1 vs. 31.89 ± 3.05). Plasma ir-inhibin and testicular ir-inhibin did not significantly change (data not shown).

### Effects of PNP on histology of the testes

**Fig. 5:**
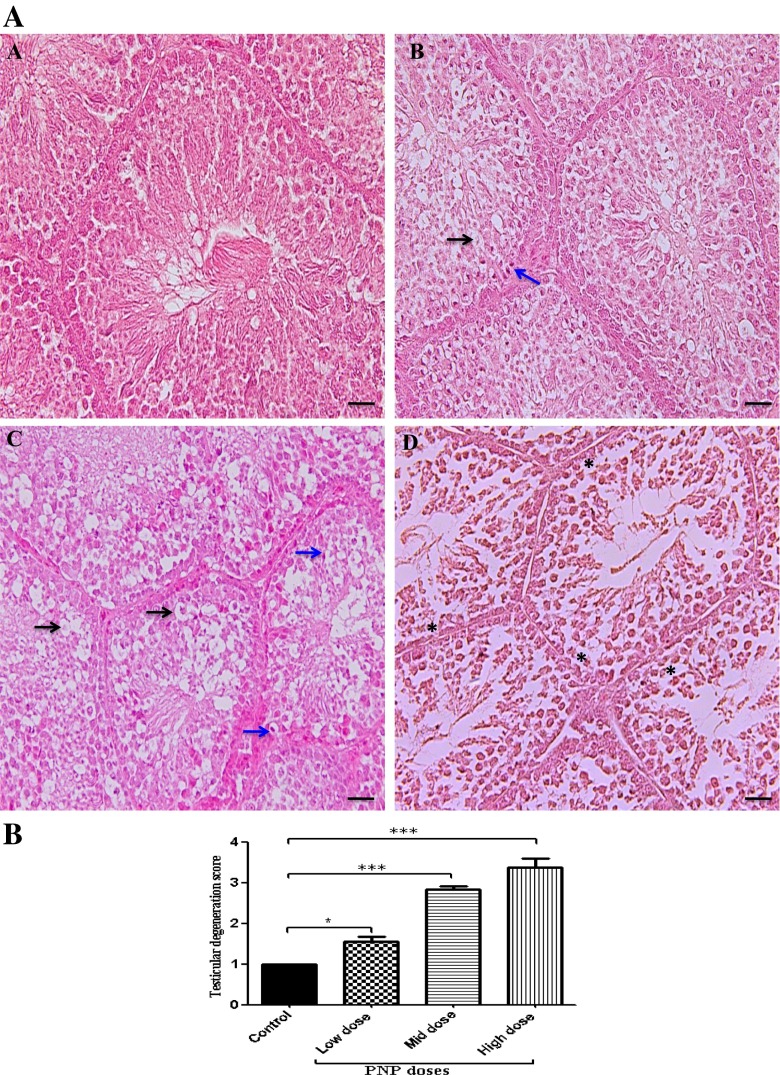
Microscopic examination (H&E stain) of the testes in the control sections (**a**) showed normal architecture and clear compartmentalization in the seminiferous tubules, with spermatozoa visible in normal-sized lumen. The PNP-treated groups; the low dose (**b**), the mid dose (**d**), and the high dose (**e**) showed vacuolation of some spermatogenic cell and spermatocyte (*black arrows*), karyopyknosis (*blue arrows*), and hypocellularity of spermatogenic cell and spermatocyte (*asterisks*), as well as cellular degeneration. The *scale bar* represents 50 μm. **b** Quantitative analysis of testicular degeneration rates in the testes of the control and the PNP-treated quails. Data are expressed as average of the scores ± S.E.M. in 250 seminiferous tubules/animal (*n* = 4). The degeneration scores ranged from normal (*1*) to mild (*2*), moderate (*3*), and severe (*4*). ***P < 0.001 in the PNP-treated groups vs. the control group

The control sections showed a normal architecture, cell association, and clear compartmentalization in the seminiferous tubules, with spermatozoa visible in normal-sized lumen (Fig. 5a (A)). However, long-term PNP exposure caused some testicular histopathological changes such as vacuolation of some spermatogenic cells and spermatocyte, karyopyknosis, and cellular degeneration as well as hypocellularity of spermatogenic cell and spermatocyte (Fig. 5a). Moreover, the score rate of testicular degeneration was very high in the PNP-treated groups (low-dose group, 1.57 ± 0.12; mid-dose group, 2.83 ± 0.09; high-dose group 3.39 ± 0.2) compared with the control group (1) as shown in Fig. 5b. A decrease of appearance of spermatids and spermatozoa population was noticed within the seminiferous tubules in the PNP high-dose (Fig. 5a, d) compared with the control group (Fig. 5a (A)).

### Immunohistochemistry of caspase-3 and claudin-1

**Fig. 6:**
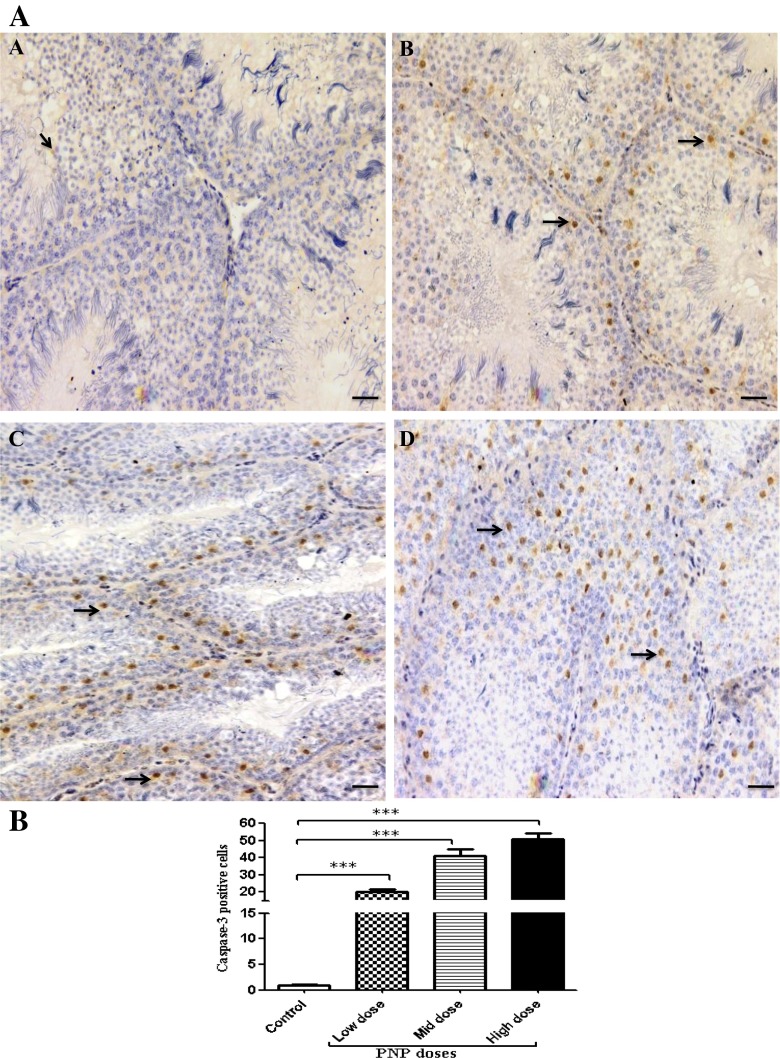
Representative caspase-3 expression in testes sections from the control (**a**) and the PNP-treated groups; the low does (**b**), the mid dose (**c**), and the high dose (**d**). The positive cells have brown nuclei (*arrows*). The main expression appears in the PNP-treated groups in the spermatogenic cell and spermatocyte. The *scale bar* represents 50 μm. **b** Quantitative analysis of caspase-3-positive cells in the testes of the control and the PNP-treated groups (*n* = 5). The positive cells were counted in 30 fields/slid. **P < 0.01; ***P < 0.001—in the PNP-treated groups vs. the control group

**Fig. 7:**
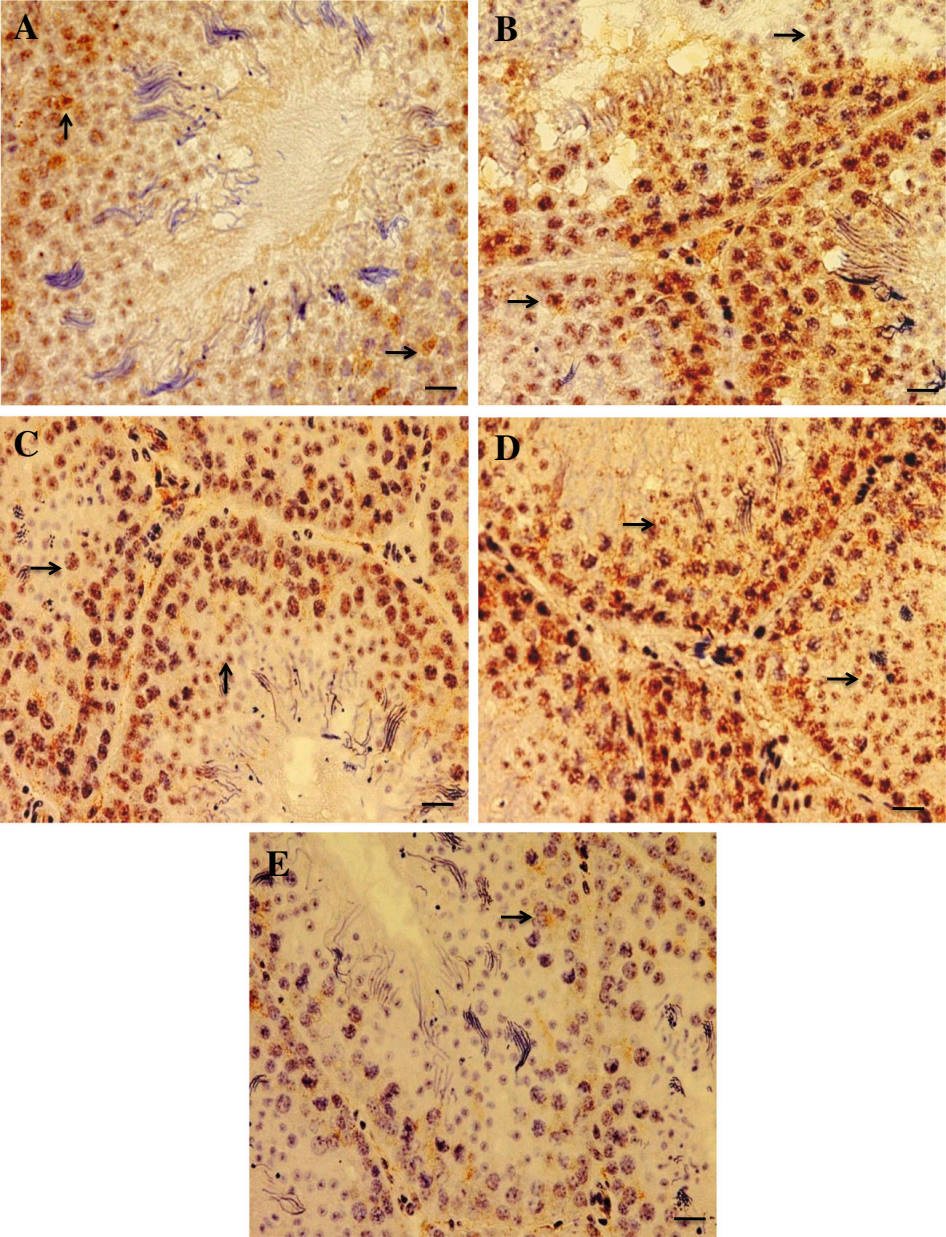
Expression of claudin-1 in testes sections from the control (**a**) and the PNP-treated groups; the low dose (**b**), the mid-dose (**c**), and the high dose (**d**, **e**). The expression appears in brown color (*arrows*). In the control group, the expression was found in early spermatocytes. In all the PNP-exposed bird, the claudin-1 expression founded widely spread in the seminiferous tubules and interstitial tissue in early and late spermatocytes. Moreover, in the high dose of PNP, some animals were showed low expression of the claudin-1 (**e**). The *scale bar* represents 20 μm

Results showed that exposure to the PNP for a long time caused activation of CASP3; apoptosis-related cysteine peptidase (low-dose group, 20.1 ± 1.7; mid-dose group, 41.2 ± 3.9; high-dose group, 50.8 ± 3.2) when compared with the control group (1.05 ± 0.2) as shown in Fig. 6a, b. The expression of CLDN1, tight junctions (TJs) proteins were disturbed in the PNP-treated groups contrary to the control birds. In the control group, CLDN1 immunoreactivity mainly expressed in spermatogonia and spermatocytes especially the primary ones (Fig. 7a). On the other hand, in the PNP-exposed birds (0.01, 0.1, and 1.0 mg/kg b.w.), CLDN1 expression found in the primary and the secondary spermatocytes, between Sertoli cells, between Sertoli cells and gonocytes, and basal membrane of Sertoli cells with widespread and strong expression in the seminiferous tubules (Fig. 7b–d). Moreover, beside the previous form in the high dose of PNP, another form of disruption appeared as some seminiferous tubules, which showed low expression level of CLDN1 even in the spermatocytes or within the Sertoli cells (Fig. 7e).

## Discussion

Accumulation of PNP, as one of the endocrine disrupting chemicals, in air (Nishioka and Lewtas 1992), soil, and water (Asman et al. 2005) could have profound deleterious effects on humans, livestock, and wildlife health through a disturbance of endocrine and reproductive systems. As far as the researchers are aware, this is the first study aimed to measure the effect of long-term low doses of PNP administration on hormonal balance, caspase-3 (CASP3), and claudin-1 (CLDN1) expressions. The present study demonstrated that PNP administration might impair the reproductive function by decreasing LH and testosterone, activating CASP3enzyme, and interfering with testis integrity through disruption of the normal distribution of CLDN1 protein.

Nonsignificant changes of quail’s body weight after 2.5 months post-PNP administration were recorded in this study. This is aligned with a previous research by Li et al. (2006a) in immature rats. These results suggested that PNP did not cause a direct toxic effect on growth. Moreover, in the current study an exponential decrease in testes weight was reported. This observation is in agreement with a previous research performed by Li et al. (2006b), which reported that similarly to PNP, 3-methyl-4-nitrophenol (PNMC; one of nitrophenol compounds in diesel exhaust particles) caused a significant testicular atrophy in Japanese quail. In other studies, estrogenic chemicals such as diethylstilbestrol and bisphenol-(A) induced testicular atrophy in chicken (Furuya et al. 2003; Rissman et al. 1984; Perrin et al. 1995).On the other hand, it was reported that PNP treatment in rats for 4 weeks did not lead to significant difference in testes weights (Zhang et al. 2013). The difference between the results among the aforementioned literature might be attributed to the difference in species sensitivity to PNP toxicity and the level of cytochrome P450 enzyme among the different species.

Cloacal gland depends upon androgen stimulation to gain and maintain its configuration during sexual development and maturation. Hence, it provides an external marker of the circulating androgen (Ball and Balthazart 2010; Kaku et al. 1993; Ottinger and Brinkley 1979). The current results showed that the cloacal gland areas were smaller in PNP-treated groups compared with those recorded in the control group. In agreement with Li et al. (2006a), PNP induced significant decreases in accessory sex organ weights at a daily dose of 0.4 mg/kg for 5 days in rats. The decrease in the cloacal gland areas in the treated may be attributed to the reduced testosterone level recorded in the present study. Moreover, it was reported that a single administration of PNMC in quails caused cloacal atrophy (Li et al. 2006b). These results supported and ascertained the ideas that PNP might have estrogenic and anti-androgenic potency, which reflected on the testes and the cloacal gland development in maturing quail. Our study is consistent with previous studies in which PNP possessed estrogenic and anti-androgenic activity in vivo and in vitro (Taneda et al. 2004; Furuta et al. 2004, 2005; Zhang et al. 2013).

Although PNP has been reported to decrease plasma LH in the current study especially after 2 and 2.5 months, the pituitary LH content did not change significantly. In another study, exposure to PNP decreased plasma LH (Li et al. 2009); however, this decrease was due to a negative feedback by the high testosterone levels. But the change in LH that was observed in the current study is likely caused by a direct effect of the PNP at hypothalamus–pituitary level through reducing either the secretion of LH-RH or the sensitivity of the anterior pituitary gland to secrete LH, which is reflected on circulating LH. The hypothalamus may be sensitive because it contains steroid-sensitive neurons, which are sensitive to hormonal substance (Sawyer and Gorski 1971; Matsumoto 1991). Previously, PNP affected the hypothalamus–pituitary–gonadal axis in the immature male rats (Li et al. 2009). In agreement with Li et al. (2006b), single PNMC administration in adult Japanese quail lowered plasma levels of LH and testosterone. The previous results indicated that the toxicity of nitrophenol compounds (PNP and PNMC) in quails is more severe than in rats.

As a result of LH decrease, testosterone decreased significantly in PNP-treated groups. This result was in consistency with those reported in quails after a single administration of PNMC (Li et al. 2006b). However, these results might be due to the decrease of circulating LH and consequently the testosterone. In contrast, previous studies showed that DEP after a single or 4 weeks injection increased the levels of serum testosterone in rats (Li et al. 2006a, 2012a). This might be attributed to defects in the metabolism of testosterone hormone and consequently, it accumulated. This hypothesis was supported by the high-level of LH. Taken together, the discrepancy of the results between the different studies might be due to different experimentation periods or different animal species.

The adrenal gland is an important endocrine organ. It secretes gluococorticoids in response to stress conditions, and thereby supports homeostasis of the organism. Moreover, the functional relationship between the gonads and the adrenal gland is well established (Li et al. 2007). In the current study, plasma corticosterone showed nonsignificant wavy changes fluctuating between decrease and increase. A previous paper reported that PNMC decreased corticosterone via a suppressive effect on adrencortical function in immature rats and adrenal cell culture (Li et al. 2007). In turn, this led to an increase in the plasma concentrations of adrenocorticotropic hormone. In addition, DEP disrupted adrenocortical function of adult male mice (Li et al. 2012b). Furthermore, the phytoestrogen genistein suppressed levels of serum cortisol in human adrenocortical cells through steroidogenic enzymes (Ohno et al. 2002). These results suggest that PNP might disrupt the adrenal function through steroidogenic enzymes because PNP has an estrogenic effect. Besides, the changes of corticosterone might be due to stress condition of PNP toxicity on the hypothalamus–pituitary–adrenal axis like PNMC (Li et al. 2007).

Histopathological results showed various degrees of testicular degeneration that may be reflected on the rate of spermatogenesis in the PNP-treated groups. These results suggested that circulating testosterone in the treated birds reduced, in addition to local toxic effects of PNP on testis. In a previous paper, after a single PNMC administration, significant reduction of spermatogenesis and a decrease of spermatozoa in seminiferous tubules were reported (Li et al. 2006b). Furthermore, injection of DEP suppressed spermatogenesis in adult mice (Li et al. 2007). In addition, our results showed that PNP caused activation of CASP3, an apoptosis-related cysteine peptidase. Likewise, in adult male mice exposed to PNP at a high dose (50 mg/kg), activation of germ cell apoptosis, Bax expression, and CASP3 enzyme activity were reported (Mi et al. 2013). In a previous study, exposure to PNMC induced spermatogenic cell apoptosis in immature male rats (Yue et al. 2011). Therefore, the results of the current study indicate that even low doses of PNP have the ability to accelerate the rate of apoptosis.

In testis, tight junctions (TJs) are important for the formation of the blood–testis barrier and crucial for spermatogenesis (Gye 2003), as well as their role in the protection of germ cells from the immune system. In regard to the CLDN1 expression, a tight junction protein, disruption in normal distribution was observed in the current study. Morrow et al. (2010) agreed with Gye (2003) that CLDN1 mRNA and protein in mouse testes decreased with age. They clarified that in a normal situation, the decrease in CLDN1 expression in testes probably represented a dilutional effect of rapid germ cell proliferation. In the same vein, Park et al. (2011) conducted a research on immature 6-week-old pheasant testis. They found that CLDN1 was found between adjacent Sertoli cells and germ cell. However, in adult pheasant testis CLDN1 was found in early spermatocytes and surrounding cytoplasm of Sertoli cells. They added that with age, a different type of tight junction protein such as claudin-11 replaced CLDN1. The results of the current study pinpointed that a disruption and a delay in the testicular development occurred, which led to the mis-distribution of the CLDN1 in the PNP-treated groups. Moreover, the testes of PNP-treated birds expressed slow rates of germ cell proliferation and immaturity resulted in an over-expression of the CLDN1. However, in the control group, the CLDN1 protein expressed in moderate intensity may be attributed to the active spermatogenesis. On the other hand, different forms of disruption occurred in the birds exposed to the high dose (1 mg/kg), which showed severe decrease in CLDN1 expression with a scanty number of spermatozoa in the lumen of seminiferous tubules when compared with the control birds which might be due to hypocellularity of spermatogenic cells and spermatocyte. Hence, this led to a loss of physical barrier and tight junction that is controlled by CLDN1.

In conclusion, PNP may have a peripheral and/or central effect. The peripheral one may be on the testicular development through induction of apoptosis-related cysteine peptidase (CASP3) and disruption of CLDN1 tight junction protein expression. The central one may be on the hypothalamic–pituitary–gonadal axis through reduction of plasma LH and consequently testosterone. Hence, it may hinder the reproductive processes. The results of the present study contribute to a further understanding of reproductive toxicity with PNP pollutant. Moreover, these results indicate that even a very small dose of the PNP has the ability to impair the reproductive process. Therefore, the possibility that large amounts of PNP in the environment will have deleterious effects on humans, livestock, and wild life cannot be ignored. To sum up, PNP should be considered as a substance clearly involved in causing deleterious effects on reproductive health.
